# A cluster randomised controlled trial protocol of an adapted intervention for alcohol use disorders in people living with HIV and AIDS: impact on alcohol use, general functional ability, quality of life and adherence to HAART

**DOI:** 10.1186/s12888-017-1208-3

**Published:** 2017-01-28

**Authors:** Munyaradzi Madhombiro, Bazondlile Dube-Marimbe, Michelle Dube, Dixon Chibanda, Moleen Zunza, Simbarashe Rusakaniko, David Stewart, Soraya Seedat

**Affiliations:** 1 Department of Psychiatry, Parirenyatwa Group of Hospitals, University of Zimbabwe, College of Health Sciences, Mazowe Street, Box A178, Avondale, Harare Zimbabwe; 2 Harare Central Hospital, Psychiatric Unit, Southerton, Harare Zimbabwe; 3 Stellenbosch University, Faculty of Medicine and Health Sciences, Biostatistics Unit, Tygerberg Campus, Parow, Cape Town, South Africa; 4 Department of Community Medicine Parirenyatwa Group of Hospitals, University of Zimbabwe, College of Health Sciences, Mazowe Street, Box A178, Avondale, Harare Zimbabwe; 5 Department of Psychology, Seattle Pacific University, 3rd Avenue W Seattle 206-281-2000, Washington, 3307 USA; 6 Department of Psychiatry, Stellenbosch University, Faculty of Medicine and Health Sciences, Tygerberg Campus, Parow, Cape Town, South Africa

**Keywords:** Alcohol use disorders, Motivational interviewing, Cognitive behavioural therapy, Intervention, Zimbabwe

## Abstract

**Background:**

Interventions for alcohol use disorders (AUDs) in HIV infected individuals have been primarily targeted at HIV risk reduction and improved antiretroviral treatment adherence. However, reduction in alcohol use is an important goal. Alcohol use affects other key factors that may influence treatment course and outcome. In this study the authors aim to administer an adapted intervention for AUDs to reduce alcohol use in people living with HIV/AIDS (PLWHA).

**Methods:**

This study is a cluster randomised controlled trial at 16 HIV care clinics. A motivational interviewing and cognitive behavioural therapy based intervention for AUDs, developed through adaptation and piloted in Zimbabwe, will be administered to PLWHA with AUDs recruited at HIV clinics. The intervention will be administered over 16 sessions at 8 HIV clinics. This intervention will be compared with an equal attention control in the form of the World Health Organization Mental Health Gap Action Programme (WHO mhGAP) guide, adapted for the Zimbabwean context. General function, quality of life, and adherence to highly active antiretroviral treatment (HAART) will be secondary outcomes. Booster sessions will be administered to both groups at 3 and 6 months respectively.

The primary outcome measure will be the Alcohol Use Disorder Identification Test (AUDIT) score. The World Health Organisation Disability Assessment Schedule 2.0 (WHODAS 2.0), World Health Organisation Quality of Life (WHOQoL) HIV, viral load, and CD4 counts will be secondary outcome measures. Outcome assessments will be administered at baseline, 3, 6, and 12 months. Moderating factors such as perceived social support, how people cope with difficult situations and post-traumatic exposure and experience will be assessed at baseline. Trained research assistants will recruit participants. The outcome assessors who will be trained in administering the outcome and moderating tools will be blinded to the treatment arms allocated to the participants. However, the principal investigator, participants and intervention staff will be unblinded.

Data will be analysed using STATA Version 14. Primary and secondary outcomes will be measured at four time points that is; at baseline, 3, 6, and 12 months respectively. All participants will be included in the analysis of primary and secondary outcome measures. The mean AUDIT scores will be compared between groups using student t-tests. Multilevel logistic regression analysis will be performed for binominal variables and multilevel linear regression for continuous variables. Descriptive statistics will be computed for baseline and follow-up assessments.

**Discussion:**

The study will be the first to address problematic alcohol use in PLWHA in Zimbabwe. It seeks to use local resources in delivering a modified, brief, evidence-based, and culturally contextualised intervention. The study results will determine the effectiveness of adapting psychological interventions for AUDs in HIV infected adults using a task-sharing framework.

**Trial registration:**

Pan African Clinical Trial Registry, PACTR201509001211149. Registered 22 July 2015.

## Background

According to the World Health Organization (WHO), alcohol abuse is one of the top three causes of health related problems apart from child underweight and unsafe sex [1]. In low and middle income countries, AUDs cause 19.5 million Disability Adjusted Life Years (DALYS) [2]. Harmful alcohol use results in 3.3 million deaths per year globally. In 2012, according to WHO alcohol use contributed 5.1% to the global burden of diseases or 139 million net DALYS. Zimbabwe is a country with one of the highest per capita alcohol consumption rates in the WHO Afro-Region at 5.7 l per capita per year in 2010 [3].

Alcohol consumption is high among people living with HIV/AIDS (PLWHA) especially hazardous alcohol consumption and this is associated with decreased survival [4]. Studies have also shown that alcohol consumption is linked to HIV infection, adherence to the highly effective antiretroviral therapy (HAART), HIV prevention, delayed testing and treatment, and general poor outcome in HIV care and treatment [5, 6]. PLWHA are at increased risk of physiologic injury such as liver disease from alcohol [7].

Heavy episodic drinking is common in sub-Saharan Africa, the epicentre of the HIV pandemic [8]. The quantity of alcohol consumed is more closely related to HIV infection than the frequency although some studies have indicated that the drinking context is as important [9]. In addition to alcohol and other drug abuse, depression, anxiety and psychosis, are linked to non-adherence to HAART and to treatment interruptions [10–12]. In order to achieve adequate viral suppression and improve adherence to HAART, AUDs and other psychiatric disorders need to be appropriately treated. Wu et al., after controlling for adherence, showed that daily consumption of alcohol was associated with a viral load increase, while reduction of alcohol intake to once weekly intake was associated with a reduction in viral load [13].

There is evidence that alcohol affects the functioning of the human immune system negatively [14]. Alcohol has been shown to increase the concentration of HIV RNA in semen and the vagina [15]. There is also some evidence that vaginal shedding of viral RNA is increased by alcohol which is associated with increased infectiousness [16].

AUDs and low adherence to HAART are associated with poor health outcomes and quality of life [17, 18], while simplification of HAART has been associated with improved adherence and quality life [19, 20]. Alcohol consumption has implications for HIV treatment through direct toxic effects on the liver and the interactions with HAART [21, 22].

Despite the high prevalence of alcohol use disorders among people living with HIV/AIDS (PLWHA) and the potential for adverse health consequences, there is not enough contextual evidence for behavioural interventions for AUDs in African settings. Motivational interviewing (MI), cognitive behavioural therapy (CBT), problem solving, and risk reduction are some of the evidence-based treatments that have been used to treat alcohol use disorders [23]. MI and CBT have been used both in combination and separately [24].

Sub-Saharan Africa suffers from the world’s most pronounced crisis in terms of human resources for health [25]. A streamlined and rationalized chain of care that relieves pressure on individual workers is needed. However this chain of care for patients, while increasing access to and uptake of interventions, must be quality assured [26]. Up skilling various cadres of staff to provide services that are normally delivered by highly skilled workers is recommended [27]. Task sharing has been proposed as an approach to provide services that are normally provided by highly skilled staff by the lesser skilled through training and provision of treatment manuals. For task-sharing to work, attention needs to be paid to the selection of staff, their current workload, the adequacy of training, and the availability of manualised interventions [28]. Supervision and support visits are essential for quality control and maintaining fidelity [29]. Task-sharing has the potential to build capacity and integrate HIV care, thereby also addressing the gap that results from health worker attrition due to immigration and HIV [30].

Most studies on HIV in Zimbabwe have focused on behavioural change with particular emphasis on risk reduction and adherence to HAART. There have been no studies of the management of AUDs among PLWHA in Zimbabwe. This is to our knowledge, the first intervention study that specifically targets AUDs in PLWHA. This intervention for AUDs intervention will be compared with the mhGAP intervention guide alcohol use management module which is a part of the WHO Mental Health Gap Action Programme (WHO mhGAP) [31]. The mhGAP intervention guide was developed for primary care settings and has been used in developing countries for the identification and management of various mental and neurological and substance use disorders (MNS).

## Methods/Design

### Preparatory-work

**Fig. 1:**
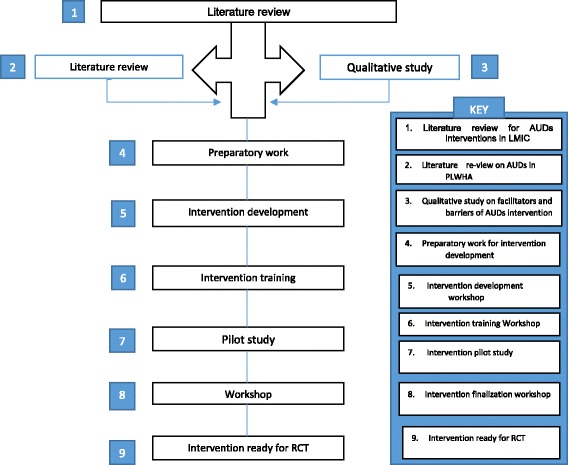
Intervention preparatory stages

In the development of the intervention for the proposed study, a literature review was undertaken to evaluate the available scientific evidence on the magnitude of the AUDs in PLWHA across Low and Medium Income Countries (LMIC). The review of literature for efficacy and effectiveness studies was focused on relevant socio-demographic variables (including gender), methods used, HIV disease progression and alcohol outcomes. This evidence was then presented to experts and HIV specialists to bring their attention to the problem and get them on board to expand on the work and bring in local context. The study flow is shown in Fig. 1.

Focus in the development of this intervention was on integration and adaptation of MI and CBT, which were the main intervention theoretical frameworks and brought in the local contextual framework. Development of treatment manuals, and exploratory work with a small number of patients then followed. The intervention development and design included meetings and workshops that were qualitative in nature. This involved experts such as provincial medical officers, district medical officers, nurses, and stakeholders from community leadership and provincial government, NGOs and alcohol service organizations such as the alcoholics anonymous and PLWHA. Interactions and formal meetings with the Ministry of Health, College of Health Sciences (Departments of Medicine, Psychiatry and Community Medicine) were done to gather their views. The National AIDS Council, which is the statutory body mandated with planning, coordinating, and evaluation of HIV/AIDS activities in Zimbabwe participated in the development of the intervention.

Focus group discussions, which were conducted with PLWHA, were around the language of alcohol and knowledge gaps concerning alcohol use. Barriers and facilitators of AUDs treatment were explored. Issues around ‘messaging’ in the intervention and patient information leaflet were discussed as well.

The current HIV clinic setup, existing staff skills in counselling and established counsellor-patient relationships was identified as the main facilitators to intervention. Stigma, lack of skills among health professionals, absence of brief culturally appropriate screening tools, and patient and provider attitudes toward their own alcohol use were identified as the main barriers. The pilot study showed that the RGNs could deliver the intervention. The recruitment and retention of participants was feasible and adjustments to the study instruments needed to be done to allow smooth flow of the study.

### Aims

1. To evaluate the effectiveness through a cluster randomized controlled trial of an adapted MI/CBT AUDs intervention in PLWHA.
2. To assess the impact of the AUDs intervention on adherence to HAART as measured by viral load and CD4 count, functional capacity as measured by the WHODAS 2.0, and quality of life as measured by the WHOQoL HIV.
3. To identify the moderators between alcohol and HIV treatment using the Cope-13, *Multidimensional Scale of Perceived Social Support (MSPSS* and the Davison Trauma Scale (DTS).

### Design

Two-arm cluster randomised controlled trial at 16 HIV clinics in Zimbabwe, with the unit of randomization HIV clinics, comparing Motivational Enhancement Therapy and Cognitive Behavioural Therapy from Project MATCH adaptation based treatment, with the WHO mhGAP intervention guide, adapted for the local context. This design has been selected to increase administrative efficiency and decrease the risk of experimental contamination at clinic level. The registered general nurses (RGNs) will be trained to administer the intervention and control using manuals.

### Study setting

The study will be conducted in HIV clinics in Zimbabwe, a sub-Saharan African country with an estimated 1,6 million PLWHA whose HIV care is decentralised. RGNs run the HIV care clinics and physicians provide supervision. RGNs provide HIV testing, adherence counseling and monitor treatment progress. The patients are reviewed, largely for reboarding of their medication. There is an electronic database of all the patients at every clinic.

### Participants

Participants are treatment-seeking adults who are HIV positive and who screen positive for AUD as per the inclusion criteria.

### Study materials

#### Socio-demographic data

Socio-demographic data such as gender, age, marital status, employment and years of education will be collected.

#### Clinical data

Clinical data such as the date of HIV testing, duration on HIV treatment, treatment regimen, CD4 count and viral load will be collected.

### Primary outcome tools

The primary outcome measures will be the change in alcohol use as measured by AUDIT score change from the baseline. Although alcohol use biomarkers would have been ideal, due to resource constraints a self-report tool will be used in this study.

#### Alcohol Use Disorder Identification Test (AUDIT) [32]

The AUDIT has not been validated in Zimbabwe, however several studies have utilized the AUDIT as an instrument to assess alcohol use in various communities including Zimbabwe [33]. In a study by Bush et al. [34] the AUDIT was found to have a sensitivity of 89% and a specificity of 67%. The unit of alcohol as defined in the AUDIT is 10 g. The legislation requires that the percentage of alcohol in alcoholic beverages be specified and follow the regional norm. The alcohol quantity in grams is however more informative. A traditional brew in the rural areas of Zimbabwe, which has 40 g of alcohol in 1.5 l, is estimated to have 4 units of alcohol and will be used to assess the quantity of units consumed according to local norms.

### Secondary outcome tools

The secondary outcomes will be the functional capacity, the quality of life and adherence to HAART.

#### World Health Organisation Disability Assessment Schedule 2.0 (WHODAS 2.0) [35]

The WHODAS 2.0 will be a secondary outcome measure and will be administered at baseline, 3, 6, and 12 months respectively to assess functional capacity. The WHODAS 2.0 contains 6 domains, which are cognition, mobility, self-care, getting along, life activities and participation. It is fairly short and simple and takes about 5–20 min to administer. Although the WHODAS 2.0 has not been validated in Zimbabwe, it has been used in HIV patients in South Africa [36].

#### World Health Organization Quality of Life (WHOQoL HIV) [37]

The WHOQoL HIV is a tool that assesses the quality of life of persons with HIV. The WHOQoL HIV has six domains that include physical, psychological, level of independence, social relationships, environment, and spirituality domains (http://www.who.int/msa/qol/). In a study to assess the overall health-related quality of life in a sample of HIV infected South Africans, results showed that quality of life, as measured by WHOQoL, was poor, with further analysis showing that the WHOQoL domains predicted overall quality of life in PLWHA [38]. Although the WHOQoL has not been validated in Zimbabwe, the Department of Psychiatry, University of Zimbabwe was involved in its development and the tool has been used in the region [39].

### Tools for confirming eligibility

The tools to confirm eligibility will assess the presence of other drug use and exclusion of participants with dementia and other psychiatric conditions.

#### Drug Use Disorders Identification Test (DUDIT) [40]

The DUDIT screens for the presence of substances of abuse other than alcohol [41]. The DUDIT has validity in determining the severity of dependency although caution needs to be exercised in deciding on cut- off points [42]. In a study in the USA the assessment of its psychometric properties, the DUDIT had a sensitivity and specificity of 90% and 80%, respectively, with an optimal cut-off of 8 [43]. The DUDIT has not been validated in Zimbabwe.

#### Substance Abuse Mental Illness Symptom Screener (SAMISS) [44]

The SAMISS identifies individuals with substance abuse and mental illness. It can be simply scored and assists in the identification of patients with probable mental illness. The SAMISS has been validated in South Africa and has been identified as a tool that can be used in primary care settings for PLWHA [45]. The SAMISS was found to have a sensitivity of 94% and a specificity of 58% and was better at identifying alcohol use (sensitivity 94% and specificity 85%) and mental illness (sensitivity 97% and specificity 60%) [45]. However, it has not been validated in Zimbabwe.

#### M.I.N.I (MINI International Neuropsychiatric Interview 7.0 [46]

The M.I.N.I for the DSM-5 is the gold standard diagnostic interview in this study and will be administered by a clinician [47]. The clinician-administered M.I.N.I. will be used to assess for common psychiatric disorders. The M.I.N.I has been recognised as a short diagnostic interview that can be easily incorporated into routine clinical assessment [48].

#### International HIV Dementia Scale (IHDS) [49]

Patients with dementia will be excluded and referred for specialist care, as they may not be able to cognitively engage with the requirements of the intervention. The IHDS has not been validated in Zimbabwe, but has been used in sub-Saharan Africa samples. In cohorts in USA and Uganda, the sensitivity and specificity using cut-off of 10 or less were 80 and 57% in the USA and 80 to 55% in Uganda [50].

### Additional assessments/ moderators

These tools assess the factors that may be related to alcohol use and HIV treatment such as history of trauma, social support and coping with stress.

#### Davidson Trauma Scale (DTS) [51]

The DTS will be used to determine the presence of traumatic event exposure and possible posttraumatic stress symptoms. HIV is associated with traumatic experiences and features of post-traumatic stress disorder (PTSD). The Davidson Trauma Scale was developed as a self-rating tool which has been shown to be useful in diagnosing and measuring symptom severity and treatment outcome in PTSD [52].

#### COPE-13 [53]

This instrument assesses how people respond to difficult or stressful events in their lives. The COPE-13 was developed to assess situational and dispositional coping styles and has been used in different samples in communities affected by natural disasters in caregivers and patients [54].

#### Multidimensional Scale of Perceived Social Support (MSPSS) [55]

The MSPSS aims to assess the social support available to the patient from significant others, family and friends. Social support can act as a buffer for psychological distress whilst the lack of it can lead to adverse outcomes such as relapse into depression and emotional distress in physical illness [56]. The MSPSS has been shown to be reliable and valid in some populations (e.g. in the Thai population) but has not been validated in Zimbabwe.

### Procedures

#### Intervention

The intervention will be blended motivational interviewing (MI) and cognitive behaviour therapy (CBT). The four key principles of MI which are to (a) express empathy, (b) develop discrepancy, (c) roll with resistance, and (d) support self-efficacy will be employed in this intervention. MI topics will help in building client motivation and emphasize responsibility for change. The provider gives guidance and support with no specific assumptions regarding the course of treatment. Cognitive behavioural therapy will include (a) identifying intrapersonal and interpersonal triggers for relapse, (b) coping-skills training, (c) alcohol refusal skills training, (d) functional analysis of alcohol use, and (e) increasing non-drinking related activities.

The intervention will have 16 sessions. Eight sessions will be given at baseline and 4 each at 3 and 6 months.

**Fig. 2:**
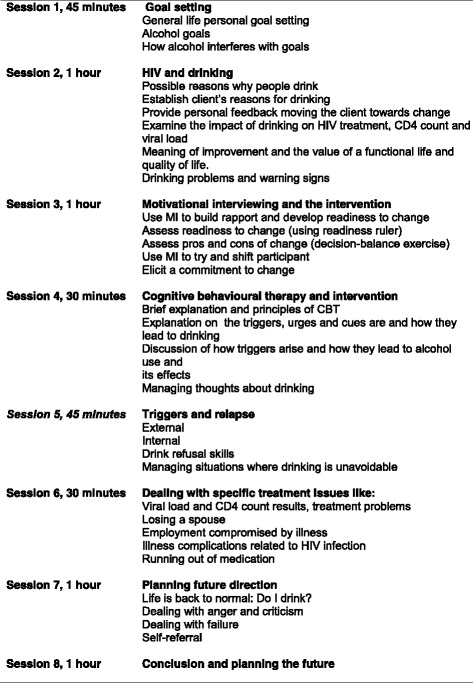
Intervention sessions, expected time and goals

Sessions 1, 4, 6 and 7 will be repeated at 3 and 6 months. Additional sessions, with content dependent on individual problems identified, will be administered. The content of the intervention is shown in Fig. 2.

#### Control

An adapted version of the mhGAP intervention guide (the alcohol section) will be administered to participants at 8 clinics. The session will also focus on providing feedback on the AUDIT score, the CD4 count, viral load, and on functioning and quality of life. This intervention is expected to last one hour (based on our experience in the pilot study). At 3 and 6 months, the control intervention will be re-administered and outcomes assessed with the final assessment done at 12 months.

#### Training

The principal investigator will be the chief trainer on the MI/CBT AUDs intervention while a co-investigator trained in the mhGAP intervention guide will train the control arm interventionists.

RGNs will be trained in the use of the intervention (MI/CBT) and control (mhGAP) manuals. Sixty-four nurses working at 16 HIV Care clinics will be trained. Material covered in the training manual will include evidence-based interventions for co-occurring HIV and AUDs, administration of the AUDIT and other data gathering tools, the theoretical basis of the intervention, counselling skills and supervision. Further, RGNs will receive training in good clinical practice, covering research ethics, the importance of maintaining confidentiality, reporting adverse events, the intervention protocol, and the process of referring patients for specialized care. Practical exercises will include self-administered quizzes, small group discussions, and case study exercises.

Training materials developed during the preparatory phase of the study will be used in the intervention and control training. RGNs who were identified and trained for the pilot study will be recruited for the training. Staff trained in the feasibility study, who are RGNs by profession, will do supervisory visits. Supervisors will use supervision protocols developed for the study. Supervisory visits will be undertaken at 3, 6 and 12 months. The ability to follow essential elements of the intervention manual as judged in a meeting of supervisors through the review of audio and videotaping will be the standard of practice. Further, trained intervention staff will be required to take a written test covering the content of the intervention and control manual and attain a pass mark of 50%.

### Participants

#### Recruitment

**Fig. 3:**
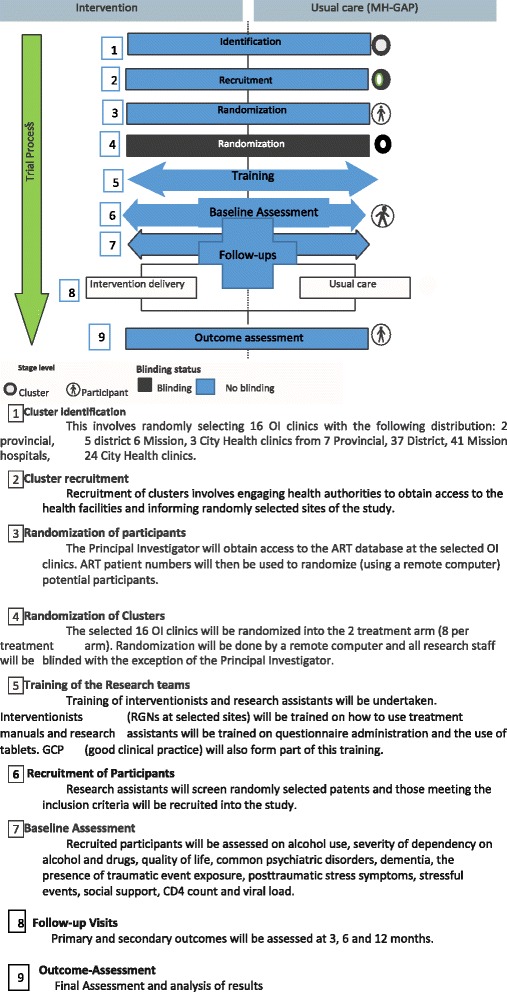
Identification of clusters and recruitment of participants

The selected 16 HIV clinics will be requested to provide patient registration numbers, which will then be entered into a remote computer to randomly select participants for screening for study eligibility. Consenting patients will be screened with the AUDIT for eligibility. A cut-off of 6 for females and 7 for males will be used [57]. The recruitment procedure and study flow are shown in Fig. 3.

#### Inclusion criteria

Study participants, aged 18 years and older, will be drawn from PLWHA on HAART receiving services from HIV care clinics in Zimbabwe. To be eligible, participants need to have been on HAART for at least three months and must be regular clinic attenders to reduce loss to follow up.

#### Exclusion criteria

Participants who are on treatment for alcohol dependency, other primary drug use disorder, and those with primary psychiatric disorder or dementia will also be excluded and referred to the psychiatrist for further assessment and care.

### Outcome measures

The primary outcome measure will be the AUDIT score. This will measure the effect of the intervention on drinking. Secondary outcome measures will comprise scores on the WHODAS 2.0, WHOQoL HIV, CD4 count and viral load. Research assistants who are graduates in health and social sciences will be trained to administer the outcome assessments. The training will include the scoring of the AUDIT, the WHODAS and the WHOQoL HIV.

### Fidelity

Fidelity has four defined components: design of the intervention, training in the intervention, monitoring the delivery of the intervention and monitoring its receipt. As it is essential to maintain fidelity, the treatment will be manualised and intervention staff will be appropriately trained to adhere to the treatment guides. Training in the intervention includes role-playing, which facilitates competence in the delivery of the intervention. As part of the training, an intervention training manual has been developed that will be used in the intervention and controls. Delivery of the intervention will be monitored through patient cards and video and audio recordings of patient session. Supervision will also be used to maintain fidelity. The standard operating procedures will contain supervision protocols. The receipt of the intervention will be monitored through patient record cards and interviews. Overall fidelity will be assessed with a comprehensive intervention fidelity guide (CIFG) [58].

### Randomization

**Fig. 4:**
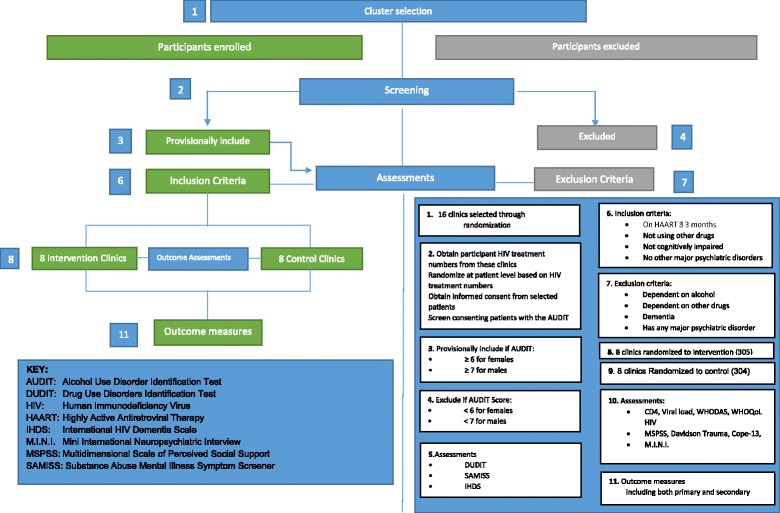
Procedure and study flow diagram

A computer generated block randomization will be used to assign clinics to either intervention or control arm (as shown in Fig. 4).

### Allocation concealment

A statistician on the study based in South Africa will maintain concealment of allocation.

### Blinding

Participants will not be blind to treatment. Research assistants who administer the outcome measures will be blinded to the assignment of clinics to intervention and control. Blinding will be achieved by retaining the same assessors at a site from the baseline, the 3,6 and 12 months assessments. In order to minimise assessment bias, assessors will not have access to the data collected at previous assessments. The Principal Investigator will be not be blinded to clinic status as he will be a trainer on the interventions.

### Visits

At the baseline visit, the AUDIT, DUDIT, SAMISS, IHDS, WHOQoL HIV and WHODAS 2 will be administered. The AUDIT, WHOQoL HIV, and WHODAS will be administered at 3, 6, and 12 months. The CD4 count and viral load will be measured at baseline, 3, 6 and 12 months to assess adherence. The MSPSS, COPE-13, and the Davison Trauma Scale will assess social support, coping and resilience at baseline and 12 months, and included as moderator variables in the analysis

### Statistical analysis

#### Sample size

A meta-analysis suggested that a clinically significant difference in negative status on the AUDIT between brief intervention and control of 13% [59]. Shersten et al. [60] describe intra-cluster correlation coefficient in human subjects to be between 0.01 and 0.02 and assumed the intra-cluster correlation in this study to be 0.02. A total of 16 clusters (8 clusters per group) to achieve 80% power to detect a mean difference of 2.5 (with precision of ± 0.45) between the treatment condition means when the standard deviation within a cluster is 4 and the intra-cluster correlation are 0.02 and a significance level of 0.05.

An estimated sample size of 180 patients (90 in the intervention group and 90 in the control group) will be required to detect a difference of 13% between intervention and control groups taking into account the design effect of 1.56. Assuming an attrition rate of 30% (as identified in the pilot study), a total sample size of 240 will be required. Fifteen participants will be randomly selected (with equal probability) at each clinic from a patient number list.

Analysis of covariance (ANCOVAs) and mixed effects linear regression models will be performed for all continuous variables. Generalised linear mixed effects logistic regression model will be performed for binary outcomes. All analyses will allow for within-clinic clustering.

### Management of missing data

Intention to treat will be the principle of statistical analysis.

### Clinic level

All clinics randomized at cluster level will be analysed by the treatment arm allocated at randomization.

### Participant level

All participants in the study will be included in the analysis of primary and secondary outcomes.

### Analysis approach

Data will be analysed using STATA Version14 [59]. Primary and secondary outcomes will be measured at four time points: baseline, 3, 6, and 12 months. To account for the stratified cluster trial design, the repeated binary and linear nature of the primary and secondary outcomes, as well as missing data at follow-up, a Generalized Estimation Equations (GEE) approach will be used. Descriptive statistics will be computed for baseline and follow-up assessments. The mean AUDIT scores will be compared using student t-tests. Multilevel logistic regression will be performed for binominal and multilevel linear regression for continuous variables.

#### Ethics

In order to promote quality control and quality assurance of the study, research staff and the principal investigator will receive training in research ethics and good clinical practise. A data research coordinator will ensure accurate data collection and the secure transfer and storage of electronic data. Weekly feedback meetings will be held with study personnel, the data manager, and the project coordinator to address problems and study-related queries.

#### Confidentiality

Participants will be assured of the confidential nature of the study. Anonymity will be maintained by de-identifying collected data and participants will be identifiable by unique identifier codes. Patient-level data will be stored securely on a computer encrypted and password protected for the study team. Files will be stored in a locked cabinet at the University of Zimbabwe College of Health Sciences, Department of Psychiatry for five years.

#### Risks and discomfort

Although no adverse effects are anticipated, participants who experience discomfort or who wish to withdraw for any other reason will be free to do so. Participants who become distressed and uncomfortable with answering the assessment questions which address sensitive topics such as alcohol use and non-adherence and when providing blood samples for CD4 counts and viral loads will be referred to a psychiatrist for further care. Participants will be free to leave the study at any point without prejudice.

#### Interim analysis

Interim statistical analysis will be carried out at 3 and 6 months. A Data and Safety Monitoring Board (DSMB) comprising of experts in HIV/AUDS, an independent statistician and a bioethicist, and the MRCZ will review study-related concerns (e.g. adverse events) that arise. They will review study protocols and procedures (e.g. data collection and storage), as they deem necessary during the data collection phase. Safeguards will be implemented throughout the project to protect participant confidentiality, as described and required by the monitoring board, and to minimize the risk of potential physical and psychological harms.

#### Costs and compensation

Participants will be reimbursed for transport costs and refreshments (US$3) for each visit.

#### Benefits

Participants may directly benefit from the interventions. Any individual identified as alcohol dependent will be referred for alcohol treatment services. Individuals assigned to the MI/CBT intervention may reduce their alcohol consumption, in turn improving HAART adherence and HIV disease outcomes. Clinic staff will benefit as they will be trained in delivering the intervention aimed at reducing harmful/hazardous alcohol consumption. Finally, substantial public health knowledge will likely be gained from this search.

## Discussion

In this study it is hypothesized that an adapted MI/CBT intervention will lead to a significant reduction in alcohol use in PLWHA. It is further hypothesized that reduction in alcohol use will lead to improved function as measured by the WHODAS 2.0, improved quality of life as measured by the WHOQoL HIV, and improved adherence to HAART as measured by the CD4 and viral load. This study further seeks to establish whether registered general nurses in primary health care facilities in Zimbabwe will be effective in providing the MI/CBT intervention to reduce alcohol use and improve patient engagement with treatment.

We will test the effectiveness of an MI/CBT intervention that can be used by RGNs for alcohol reduction in PLWHA. If successful, the intervention will be implemented in HIV clinics in Zimbabwe and other settings where general nurses oversee HIV treatment. We hypothesize that patients may view their ability to return to work as important and this may motivate them to adhere to the treatment. Improved quality of life has been shown to improve adherence in other studies.

Given that most behavioural interventions for alcohol use have been developed within a Western framework, the use of an adapted intervention in this study will inform us about its effectiveness in reducing alcohol use in PLWHA in a resource limited setting. In resource rich environments, inpatient and outpatient services for addictions are usually widely available. However, in Zimbabwe, as in many other resource-limited settings, inpatient management of AUDs may be unaffordable for the majority. Outpatient care, if available, is the rule rather than the exception.

To the authors’ knowledge, no published study from Zimbabwe has examined functioning and quality of life in PLWHA with AUDs. Improvement in functioning and quality of life are key aspects to address in this population. Quality of life has been shown to improve upon initiation of HAART (http://www.stata.com) and studies in other countries have documented an improvement in adherence with an improvement in quality of life [61]. Over a quarter of 15–49 year olds in sub-Saharan Africa are living with AIDS [62]. Given that this is the most productive age group in the community, improving functioning will likely have positive socio-economic impacts on the community.

There are few limitations that warrant mention. Firstly, we will use a self-report screening measure of drinking. Self-report may not accurately reveal drinking quantity and behaviour. That said, the AUDIT has excellent sensitivity and specificity and cross-cultural applicability and can be used in remote areas where sampling for alcohol biomarkers may be challenging. Secondly, brief interventions have been shown to be more useful when provided at community level although the lack training often hampers provision at community level. Thirdly, viral suppression may not correlate well with adherence; even in resource rich settings viral suppression can be low despite an adherence of 80–95%. In this study viral load will be a proxy measure of adherence. CD4 count in addition to the viral load will be used as a measure of adherence.

This is a task-sharing intervention study. It is hoped that, upon successful completion of this study, RGNs from district hospitals and municipal health facilities will be trained in recognising and managing AUDs in HIV infected patients.

## Abbreviations

AIDS: Acquired immunodeficiency syndrome
ANCOVA: Analysis of covariance
AUDIT: Alcohol use disorder identification test
CBT: Cognitive behavioural therapy
CIFG: Comprehensive Intervention fidelity guide
DSMB: Data monitoring and safety board
DTS: Davidson trauma scale
DUDIT: Drug use disorders identification test
FGD: Focus Group Discussion
GEE: Generalized estimation equations
HAART: Highly active antiretroviral therapy
HIV: Human immunodeficiency virus
IHDS: International HIV dementia scale
M.I.N.I.: Mini International neuropsychiatric interview
mhGAP: Mental health gap action programme
MI/CBT: Motivational interviewing/cognitive behavioural therapy
MSPSS: Multidimensional scale of perceived social support
PLWHA: People living with HIV/AIDS
PTSD: Post-traumatic stress disorder
RCT: Randomised controlled trial
RGN: Registered general nurses
SAMISS: Substance abuse mental illness symptom screener
WHO: World Health Organisation
WHODAS: World Health Organisation Disability Assessment Schedule
WHOQoL: World Health Organisation Quality of Life
